# Cancer and Cardiovascular Disease

**DOI:** 10.4061/2011/943748

**Published:** 2011-08-25

**Authors:** Syed Wamique Yusuf, Carlo Cipolla, Jean-Bernard Durand, Daniel J. Lenihan

**Affiliations:** ^1^Department of Cardiology, The University of Texas MD Anderson Cancer Center, 1515 Holcombe Blvd., Unit 1451, Houston, TX 77030, USA; ^2^Cardiology Unit, European Institute of Oncology, Via Ripamonti 435, 20141 Milan, Italy; ^3^Division of Cardivascular Medicine, Vanderbilt University, 1215 21th Avenue South, Nashville, TN 37232, USA

Cancer and cardiovascular disease (CVD) are the two most common causes of mortality and morbidity worldwide. The incidence of both cancer and cardiovascular disease increases with age. With increased life expectancy, the burden of both these diseases will increase substantially over the next generation. Advancement in cancer therapy and supportive care has led to increasing number of survivors of childhood cancer. Seventy percent of the children diagnosed with malignancy before the age of 15 years will have a disease-free 5-year survival from diagnosis. Cancer is now being recognized as a chronic disease as evidenced by a growing number of cancer survivors that currently exceeds 11 million. With further improvement in cancer therapy, this number will likely increase in years to come. As the numbers of survivors grow so does the number of patients living with the late effects of cancer-related cardiotoxicity. Amongst Hodgkin lymphoma patients who have received radiation, CVD is one of the most common causes of death.

Physicians and ancillary staff frequently have to take care of patients with concomitant cancer and cardiovascular disease. Some cardiac diseases predates the diagnosis of cancer, whereas other conditions like chemotherapy-induced cardiomyopathy and radiation-related heart disease are directly related to the cardiotoxic side effects of cancer therapy. The cardiotoxic side effects of agents like 5-fluouracil, adriamycin, and tyrosine kinase inhibitors are well known. However, the cardiotoxic profiles of newer investigational chemotherapeutic agents are largely unknown.

Chemotherapy frequently induces thrombocytopenia, which in itself poses therapeutic challenge in the management of conditions like acute coronary syndrome, atrial fibrillation, stroke, and prosthetic valves. Evidence-based treatment of cardiovascular disease in cancer patients is lacking largely because all cardiology trials have excluded patients with cancer and similarly cancer trials have excluded patients with significant cardiovascular comorbidity. 

While recently some single-center studies have shown the efficacy of medications like ace inhibitors and beta blockers for the treatment of chemotherapy-induced cardiomyopathy, evidence-based treatment of other major cardiovascular diseases in cancer patients is not well established.

In this issue of cancer and cardiovascular disease, we have covered some common conditions like venous thrombosis, cardiovascular effects of radiation therapy, cardiovascular effects of anthracycline in childhood cancer survivors, and management of aortic aneurysm in cancer patients. The use of newer modality, like computed tomographic angiography, may provide a pivotal role in the investigation of cancer patients with concomitant cardiac problem, as outlined in a clinical investigation in this journal. The case reports presented are some conditions that are unique to cancer population. Cardio-oncology is a growing field, and we are encouraged by the number of submissions to our special edition. We hope that the readers will find this special edition useful in their clinical practice.

*Syed Wamique Yusuf* *Carlo Cipolla* *Jean-Bernard Durand* *Daniel J. Lenihan*

